# Risk of interstitial lung disease in non-small cell lung cancer treated with EGFR-TKI: a real-world pharmacovigilance study

**DOI:** 10.3389/fphar.2025.1652750

**Published:** 2025-08-29

**Authors:** Xu Miao, Yuan Liu, Xiaoqin Li, Rong Zhao

**Affiliations:** ^1^ Department of Pharmacy, Affiliated Hospital of Jiangsu University, Zhenjiang, Jiangsu, China; ^2^ Adverse Drug Reaction Monitoring Department, Food and Drug Supervision and Inspection Center in Zhenjiang, Zhenjiang, Jiangsu, China; ^3^ Oncology Department, Affiliated Hospital of Jiangsu University, Zhenjiang, Jiangsu, China

**Keywords:** FDA adverse event reporting system, EGFR-TKI, interstitial lung disease, pharmacovigilance, adverse events

## Abstract

**Background:**

Epidermal growth factor receptor tyrosine kinase inhibitors (EGFR-TKIs) have emerged as a mainstay for patients diagnosed with non-small cell lung cancer (NSCLC). However, interstitial lung disease (ILD), potentially fatal, may develop in certain patients during EGFR-TKI therapy. We aimed to characterize EGFR-TKI-associated ILD and examine the risk factors.

**Methods:**

Adverse event (AE) reports from the FDA Adverse Event Reporting System were retrieved from Q1 2004 to Q1 2024. AEs were identified at the preferred term level using the Standardized MedDRA Query. Four disproportionality analyses were conducted to quantify the signal of ILD associated with EGFR-TKIs. The risk of ILD was subsequently analyzed using multifactorial logistic regression.

**Results:**

A total of 20,195 EGFR-TKI-related AE reports were analyzed, with 660 cases linked to ILD. osimertinib accounted for the most ILD reports (156), while dacomitinib showed the highest reporting odds. Subgroup analyses revealed distinct pulmonary toxicity profiles across the different EGFR-TKIs. Erlotinib exhibited the longest median time to onset. Older age, concomitant dyslipidemia, and concomitant use of lansoprazole significantly increased the risk.

**Conclusion:**

ILD risk is elevated in EGFR-TKI-treated NSCLC patients, particularly with older age, comorbidities, and lansoprazole use. Clinicians should consider these factors to reduce ILD incidence.

## 1 Introduction

Lung cancer is the leading cause of cancer-related deaths, with a 5-year survival rate of only 5%, of which Non-Small Cell Lung Cancer (NSCLC) accounts for 85% (Sung et al., 2021). Despite significant therapeutic advances over the past decade, NSCLC remains incurable for many patients (He et al., 2021). The epidermal growth factor receptor (EGFR) is a common driver gene in NSCLC, and approximately 20% of patients harbor activating EGFR mutations, making them targets for EGFR Tyrosine Kinase Inhibitors (EGFR-TKIs) (John et al., 2022; Roskoski, 2019). Current guidelines recommend EGFR-TKIs as first-line therapy for these advanced patients.

First-generation EGFR-TKIs (gefitinib, erlotinib) have been widely used, with gefitinib approved by the FDA as first-line treatment for advanced NSCLC patients with activating EGFR mutations (Kazandjian et al., 2016). Multiple studies have shown that gefitinib significantly prolongs progression-free survival in patients with advanced NSCLC (Zhang et al., 2012; Rosell et al., 2012). However, approximately 30% of patients exhibit primary or acquired resistance (Ohashi et al., 2013). While second-generation agents (afatinib, dacomitinib) have extended PFS and overall survival (34.1 months vs. 27.0 months) (Mok et al., 2021), they are associated with higher rates of cutaneous and gastrointestinal toxicity (He et al., 2021). Third-generation drugs, represented by osimertinib, have demonstrated improved efficacy while reducing the risk of severe adverse events (Soria et al., 2018) and show significant efficacy against brain metastases (Popat et al., 2023).

Although EGFR-TKIs have proven efficacy, their associated adverse reactions require careful attention, particularly interstitial lung disease (ILD). This fatal condition accounts for 58% of EGFR-TKI treatment-related deaths (Ohmori et al., 2021). Studies have reported cases of ILD in patients treated with gefitinib, erlotinib, and afatinib (Kashiwabara et al., 2017). Identifying and managing the risk of ILD is a significant clinical challenge. Traditional clinical trials, limited by factors such as sample selection, often fail to reflect the true risk in the patient population. Therefore, large samples, real-world studies are necessary to evaluate the risk characteristics of ILD.

Currently, there has been some exploration into the risk factors for EGFR-TKI-associated ILD. A study including 89 NSCLC patients aged 75 and older showed that the incidence of ILD was significantly higher in elderly patients treated with osimertinib (Ishibashi et al., 2023). However, this association and the specific risk threshold lack validation from large-sample, real-world data. Regarding dyslipidemia, existing research suggests that lipid metabolism disorders may contribute to the process of pulmonary fibrosis by promoting chronic inflammation and oxidative stress in the lungs. Furthermore, a synergistic effect of dyslipidemia has been observed in ILD induced by other drugs (Wu et al., 2023). However, research on the interaction between EGFR-TKIs and dyslipidemia is limited, and whether a dose-effect relationship exists between the two remains unclear. Additionally, some studies have indicated that the risk of developing ILD increases in NSCLC patients who use EGFR-TKIs in combination with proton pump inhibitors (Wang et al., 2025). Nevertheless, research on the impact of concomitant use of EGFR-TKIs with other drugs on ILD is currently lacking.

This study conducted a retrospective pharmacovigilance analysis of EGFR-TKIs using the FDA Adverse Event Reporting System (FAERS) database, aiming to elucidate the risk and characteristics of ILD associated with EGFR-TKI use, thereby providing clinicians with more reliable risk management strategies that could improve treatment outcomes and reduce adverse events in patients.

## 2 Patients and methods

### 2.1 Data source

This pharmacovigilance study utilized the FAERS database to investigate the correlation between EGFR-TKIs and ILD. FAERS collects post-marketing adverse event (AE) reports for drugs and biologics submitted by healthcare professionals, consumers, and manufacturers, comprising seven linked subfiles (e.g., demographics, drug info, adverse events) share a common PRIMARYID.

**FIGURE 1:**
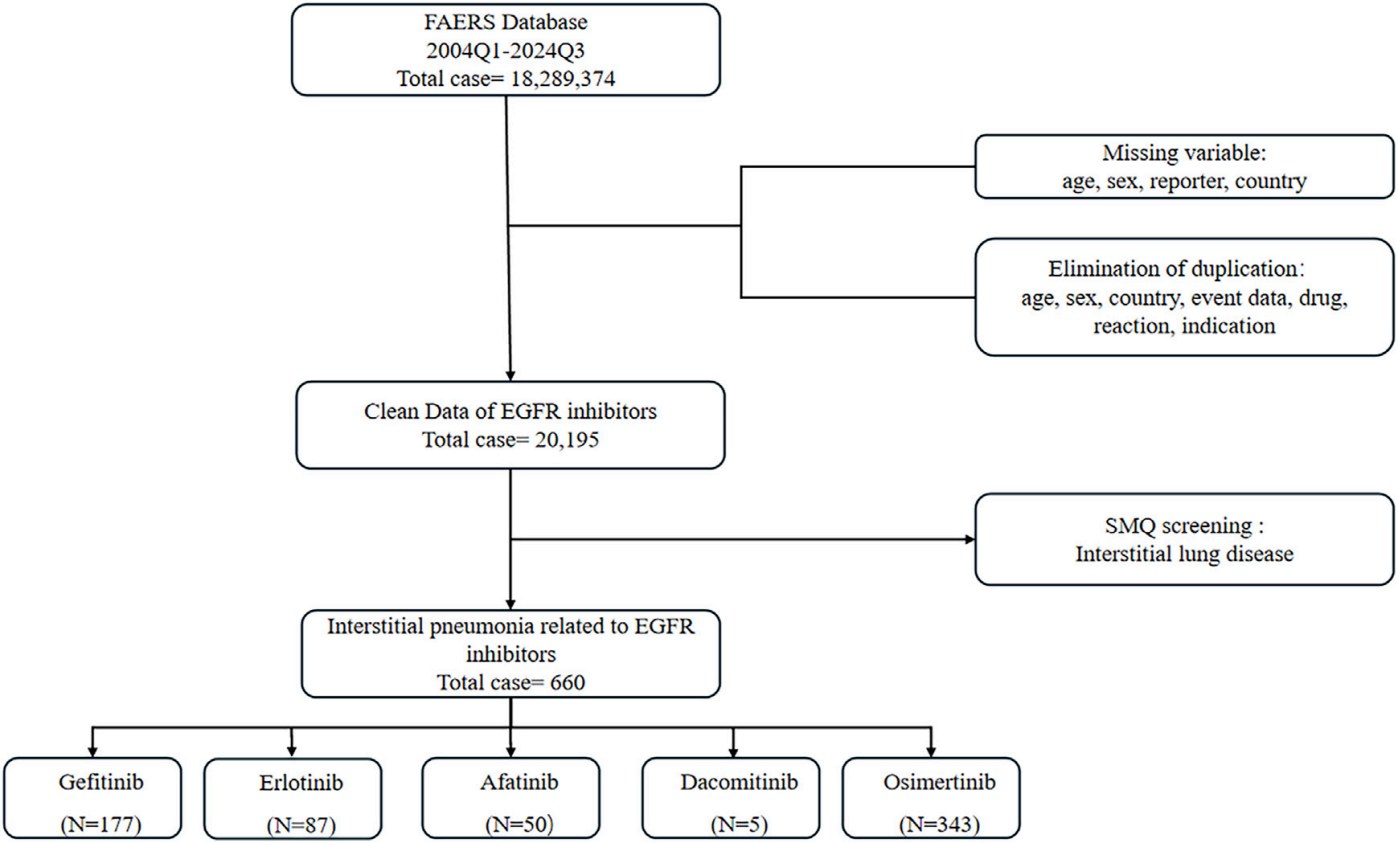
Incorporation criteria flowchart.

We analyzed the ASCII data files from the first quarter (Q1) of 2004 to the fourth quarter (Q4) of 2024. In accordance with the U.S. FDA recommendations, duplicate reports were removed by retaining the most recent case number (Sakaeda et al., 2013). Concurrently, cases with missing information on age, sex, reporter, or country were excluded, as were records containing duplicate entries for age, sex, country, drug, event, outcome, and comorbidities. Following this screening process, we identified 20,195 unique reports from the initial 18,289,374 records. Among these, 660 reports were associated with ILD. The distribution of these ILD reports across specific EGFR-TKIs was as follows: gefitinib (*n =* 177), erlotinib (*n =* 87), afatinib (*n =* 50), dacomitinib (*n =* 5), and osimertinib (*n =* 343) (Figure 1). It is critical to acknowledge inherent limitations of FAERS, including underreporting (especially for mild events), potential reporting biases (towards severe outcomes), and the absence of denominator data precluding incidence rate calculation. These necessitate cautious interpretation of disproportional signals.

### 2.2 Drug identification

Drugs in the FAERS database were submitted without a standardized approach. To obtain a comprehensive understanding of the relevance of EGFR-TKIs to ILD, both generic and brand names of EGFR-TKIs were used to extract the data (Supplementary Table S1). The inclusion criteria were restricted to drugs approved by the FDA within the study’s timeframe to ensure temporal relevance.

### 2.3 Data mining

The Medical Dictionary for Regulatory Activities (MedDRA) is a standardized medical terminology commonly used to code adverse reactions in spontaneous pharmacovigilance reports and clinical study reports (MedDRA, 2016). In this study, we used MedDRA (version 25.0) to screen the preferred terminology (PT) related to ILD (20000042) using a standardized MedDRA query (SMQ). Supplementary Table S2 systematically compiles a list of PTs related to clinical conditions associated with ILD.

### 2.4 Adverse drug reaction (ADR) signal detection

In this study, the disproportionate method was used to analyze ADR signals, which mainly includes the reporting odds ratio (ROR) method, the proportional reporting ratio (PRR) method, the Bayesian confidence interval propagation neural network (BCPNN) method, and the multi-item gamma Poisson shrinker (EBGM) method. Non-Bayesian methods (e.g., ROR) may show better efficacy in early signal detection, whereas Bayesian methods are more adept at detecting unique signals, which can be effectively identified even when fewer ADR events are reported for the drug. Supplementary Table S3 provides a 2 × 2 league table with the detailed formulas and positive-signal thresholds for these proportional imbalance analysis methods. Specifically, when the ROR method shows ≥3 reports and the lower limit of the 95% confidence interval (CI) is ≥ 1, a risk signal is detected. A risk signal is also detected when PRR ≥2 and *χ* ^*2*^ ≥ 4. The signal detection index for the BCPNN method is the information component (IC) value, and the condition for generating a positive signal is that the lower limit of the IC (IC025) is >0 (Shu et al., 2022). To confirm the generation of a valid signal, this study requires that all the above conditions be met simultaneously.

### 2.5 Statistical analysis

The clinical characteristics of retrieved patients with EGFR-TKI-related ILD were analyzed descriptively. Frequencies and percentages were used for categorical variables. The median and interquartile range were used for continuous variables. All analyses were performed using R (v 4.4.1) software, and *P* values less than 0.05 were considered statistically significant.

## 3 Results

### 3.1 Basic information

The study found that adverse reactions leading to ILD with EGFR-AKI occurred primarily in the adult population, with a particular concentration in the 65–85-year-old age group osimertinib (156 cases) and gefitinib (76 cases) were the most reported.

Regarding gender, a higher proportion of male patients developed ILD with gefitinib, erlotinib, and afatinib, whereas a higher proportion of female patients were treated with dacomitinib and osimertinib, particularly osimertinib. Regarding body weight, most patients in most drug treatment groups were clustered in the 50–100 kg range, although there was a high level of missing data. The osimertinib group had the largest number of patients weighing between 50 and 100 kg, totaling 136 patients. Most reports were submitted by physicians, with the highest number of reports in the osimertinib group (264 cases). Patient outcomes were predominantly hospitalization and death, reflecting a more severe, progressive course. Regarding the patients’ countries, the proportion of Japanese patients was 47.1% in the erlotinib group, compared with 81.9% in the osimertinib group. In contrast, Chinese patients were underrepresented in the gefitinib group, while U.S. patients were more represented in the erlotinib group (47.1%) (Table 1).

**TABLE 1:** Demographics related to ILD reported in patients receiving EGFR-TKIs.

Variable	Gefitinib	Erlotinib	Afatinib	Dacomitinib	Osimertinib
	(*N =* 177)	(*N =* 87)	(*N =* 50)	(*N =* 5)	(*N =* 343)
Sex, n (%)					
Female	67 (37.9%)	30 (34.5%)	22 (44.0%)	2 (40.0%)	204 (59.5%)
Male	110 (62.1%)	57 (65.5%)	28 (56.0%)	1 (20.0%)	135 (39.4%)
Missing	0	0		2 (40.0%)	4 (1.2%)
Weight (kg), mean ± SD					
<50 kg	25 (14.1%)	7 (8.0%)	1 (2.0%)	0	128 (37.3%)
50–100 kg	53 (29.9%)	30 (34.5%)	29 (58.0%)	3 (60.0%)	136 (39.7%)
>100 kg	0	5 (5.7%)	3 (6.0%)	0	1 (0.3%)
Missing	99 (55.9%)	45 (51.7%)	17 (34.0%)	2 (40.0%)	78 (22.7%)
Age (years), mean ± SD					
≤17	0	1 (1.1%)	0	0	0
18–64	37 (20.9%)	31 (35.6%)	17 (34.0%)	1 (20.0%)	46 (13.4%)
65–85	76 (42.9%)	44 (50.6%)	31 (62.0%)	2 (40.0%)	156 (45.5%)
≥86	5 (2.8%)	1 (1.1%)	0	0	14 (4.1%)
Missing	59 (33.3%)	10 (11.5%)	2 (4.0%)	2 (40.0%)	127 (37.0%)
Report source, n (%)					
CN	1 (0.6%)	13 (14.9%)	3 (6.0%)	0	13 (3.8%)
HP	1 (0.6%)	0	1 (2.0%)	1 (20.0%)	8 (2.3%)
MD	98 (55.4%)	42 (48.3%)	39 (78.0%)	4 (80.0%)	264 (77.0%)
OT	5 (2.8%)	27 (31.0%)	1 (2.0%)	0	9 (2.6%)
PH	7 (4.0%)	5 (5.7%)	3 (6.0%)	0	22 (6.4%)
Missing	65 (36.7%)	0	3 (6.0%)	0	27 (7.9%)
Outcome, n (%)					
DE	93 (52.5%)	35 (40.2%)	26 (52%)	0	164 (47.8%)
DS	4 (2.3%)	1 (1.1%)	0	0	0
HO	44 (24.9%)	34 (39.1%)	15 (30%)	4 (80.0%)	109 (31.8%)
LT	23 (13.0%)	5 (5.7%)	5 (10%)	1 (20.0%)	24 (7.0%)
OT	7 (4.0%)	11 (12.6%)	3 (6%)		38 (11.1%)
RI	6 (3.4%)	0	0	0	0
Missing	0	1 (1.1%)	1 (2%)	0	8 (2.3%)
Country, n (%)					
China	4 (2.3%)	0	1 (2.0%)	0	6 (1.7%)
Japan	53 (29.9%)	18 (20.7%)	21 (42.0%)	0	281 (81.9%)
United States	11 (6.2%)	41 (47.1%)	7 (14.0%)	1 (20.0%)	19 (5.5%)
Others	120 (67.8%)	28 (32.2%)	21 (42%)	4 (80%)	37 (10.8%)

CN, consumer; HP, health professional; MD, medical doctor; PH, pharmacist; OT, other; DE, death; DS, disability; HO, hospitalization; LT, life-threatening; OT, other serious outcomes; RI, required intervention.

**FIGURE 2:**
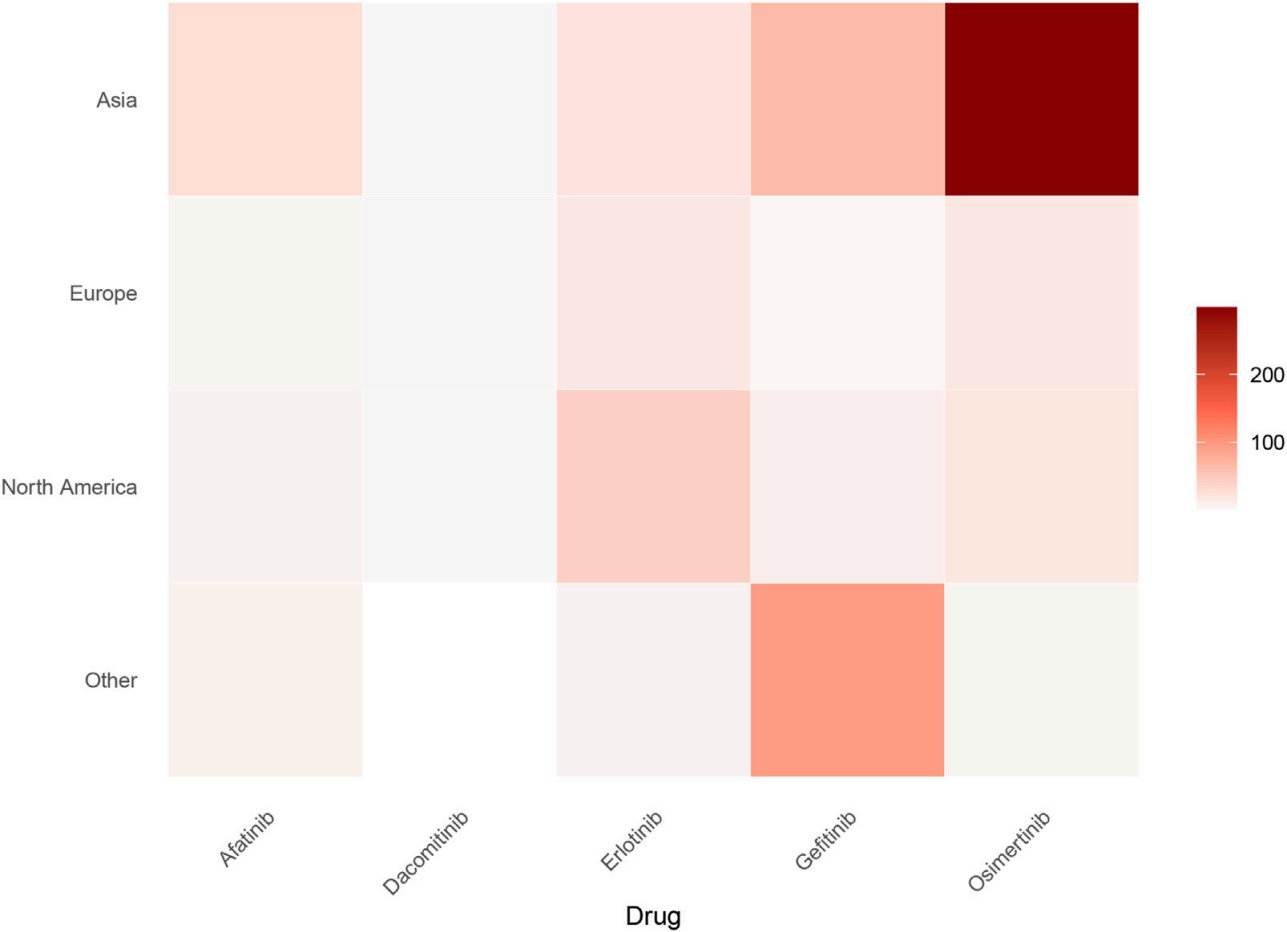
Geographic distribution of ILD cases associated with five EGFR-TKIs (absolute numbers). Heatmap depicts the absolute number of ILD cases reported per drug across major global regions (Asia, Europe, North America, Others). Color intensity scales with case counts, with darker hues indicating higher numbers. Osimertinib shows the highest burden in Asia, while gefitinib predominates in other regions.

This analysis incorporates drug utilization data across major global regions, including Asia, Europe, North America and other regions. Based on the geographic region of the patient, we generated a heatmap of the number of patients associated with five anti-EGFR drugs. As depicted in Figure 2, osimertinib demonstrated the highest case frequency in Asian populations, while gefitinib predominated in other regions. These findings suggest significantly higher anti-EGFR therapy utilization rates in Asian populations compared to other geographic regions.

### 3.2 Disproportionality analysis

As shown in Table 2, ILD signals were detected for all EGFR-TKIs. Treatment with EGFR-TKIs was significantly associated with ILD with a ROR of 24.61 (95% CI: 21.65–27.98), a PRR of 8.94, an IC: 3.03 (IC025: 1.36–4.7) and an EBGM of 8.19 (95% CI: 7.35–9.11). Dacomitinib reported the highest ROR for ILD at 21.39 (95% CI: 5.74–79.68), though this signal was derived from only 5 ILD cases, requiring cautious interpretation due to limited statistical reliability. followed by afatinib (14.93, 95% CI: 10.13–21.98) and erlotinib (12.62, 95% CI: 9.55–16.68).

**TABLE 2:** Signal detection of ILD for each EGFR inhibitor at the SMQ level.

PT	ROR (95%Cl)	PRR (*χ* ^*2*^)	EBGM (95%CI)	IC (IC_025_)
Gefitinib	12.52 (10.26–15.28)	6.75 (1014.31)	6.58 (5.57–7.78)	2.72 (1.05–4.39)
Erlotinib	12.62 (9.55–16.68)	6.78 (525.08)	6.7 (5.3–8.46)	2.74 (1.06–4.42)
Afatinib	14.93 (10.13–21.98)	7.9 (331.74)	7.83 (5.67–10.83)	2.97 (1.28–4.66)
Dacomitinib	21.39 (5.74–79.68)	10.06 (43.15)	10.05 (3.35–30.21)	3.33 (1.43–5.23)
Osimertinib	12.5 (10.79–14.48)	6.74 (1842.13)	6.45 (5.7–7.3)	2.69 (1.02–4.36)
EGFR-TKIs	24.61 (21.65–27.98)	8.94 (5000.28)	8.19 (7.35–9.11)	3.03 (1.36–4.7)

**FIGURE 3:**
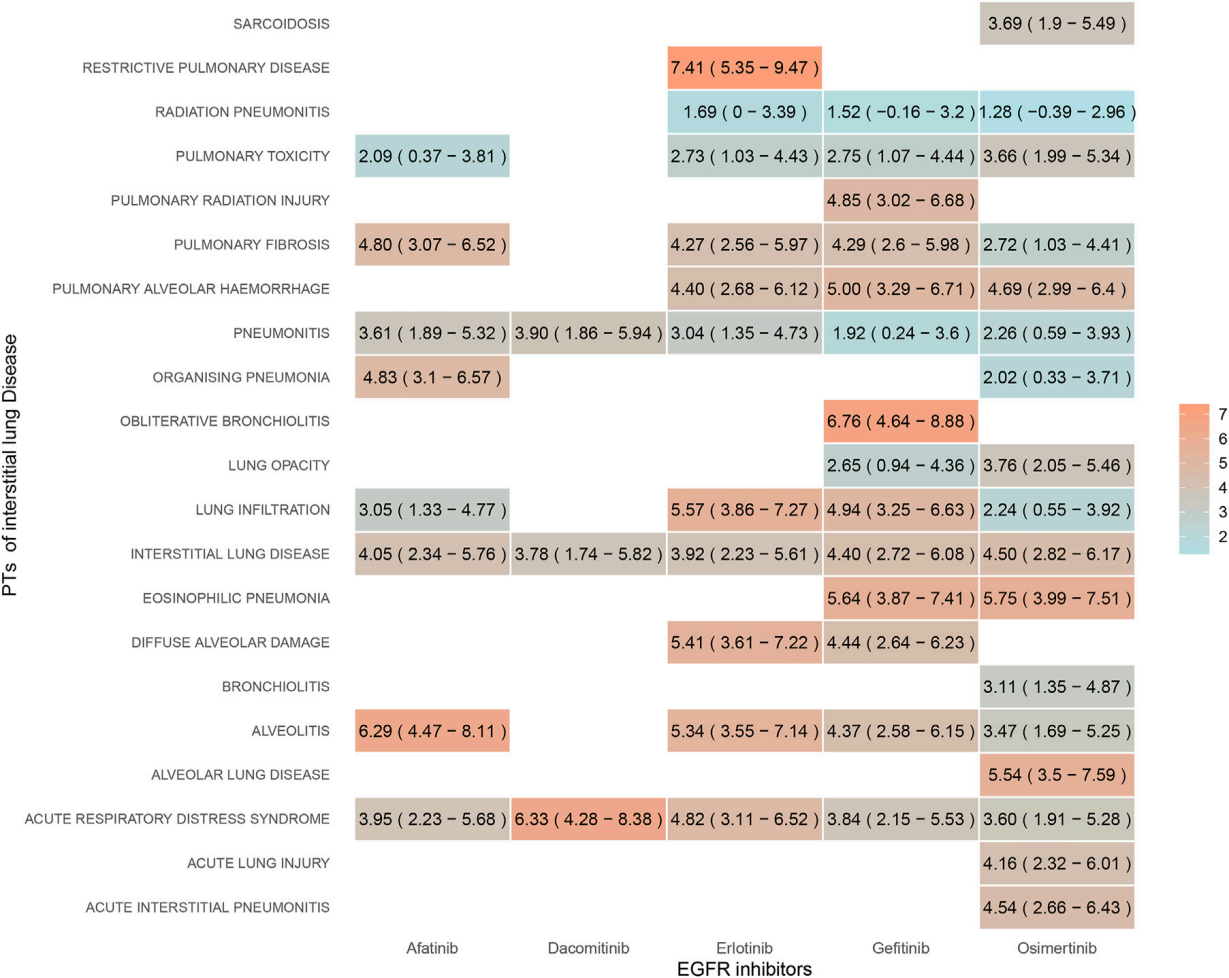
Signal strength of ILD subtypes for EGFR-TKIs quantified by reporting odds ratio. Color gradient reflects reporting odds ratio values (darker = stronger association). Osimertinib shows broadest toxicity profile (sarcoidosis, bronchiolitis), while gefitinib uniquely links to radiation lung injury.

Disproportionality analysis of the ILD subgroup revealed that 21 PTs were significantly associated with EGFR-TKIs overall. Figure 3 illustrates the strength of the association between the five EGFR-TKIs and various PTs using color shades, with darker colors indicating stronger associations. Seven PTs were significantly associated with afatinib, three with dacomitinib, and ten and eleven with erlotinib and gefitinib. Thirteen PTs were associated with osimertinib. Our results identified significant signals for interstitial lung disease and acute respiratory distress syndrome across all EGFR-TKIs. All EGFR-TKIs except dacomitinib had significant signals for lung infiltration, pulmonary toxicity, pulmonary fibrosis, and alveolitis. Only osimertinib was associated with sarcoidosis, bronchiolitis, alveolar lung disease, acute lung injury, and acute interstitial pneumonitis. Pulmonary radiation injury and signaling of obliterative bronchiolitis were observed only with gefitinib.

### 3.3 Time of onset of EGFR-TKI-associated ILD

**FIGURE 4:**
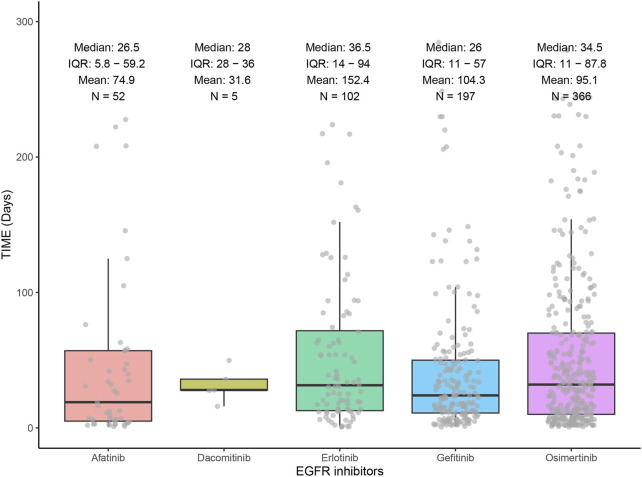
Time to onset of interstitial lung disease induced by EGFR inhibitors.

Figure 4 demonstrates the distribution of ILD onset times for the five EGFR-TKIs through a combination of box-and-line and scatter plots. Erlotinib had the longest median onset time of 36.5 days, followed by osimertinib with a median onset time of 34.5 days, and gefitinib with the shortest median onset time of 26 days.

### 3.4 Risk factors for developing ILD

Patients treated with EGFR-TKIs were analyzed for ILD risk factors. As shown in Table 3, the results of univariate logistic regression indicated that older age, female gender, concomitant diseases, and concomitant medications significantly increased the risk of developing ILD (*P* < 0.05). Conversely, weight gain was negatively associated with the risk of ILD (OR = 0.97, 95% CI: 0.96–0.98). To further explore the effect of concomitant factors on the occurrence of ILD, we observed that patients with hypertension, dyslipidemia, gastric disorders, constipation, and pain might be at higher risk than those without concomitant diseases. Additionally, the risk of ILD associated with EGFR-TKI combined with amlodipine, lansoprazole, and magnesium oxide was higher than that of EGFR-TKI monotherapy. According to multifactorial logistic regression analysis adjusting for confounding variables, as shown in Table 4, older age, concomitant dyslipidemia, and lansoprazole had a significant effect on the occurrence of ILD in patients receiving EGFR-TKIs (*P* < 0.05). Weight gain reduces the risk of developing ILD (*P* < 0.001).

**TABLE 3:** One-way logistic analysis of risk of inducing ILD.

Variable	Bate	SE	Z	*p*	OR (95% CI)
Age	0.02	<0.01	4.02	<0.001	1.02 (1.01–1.03)
Weight	<0.01	<0.01	<0.01	<0.001	0.97 (0.96–0.98)
Sex	2.64	0.41	6.4	<0.001	14.05 (6.26–31.54)
Concomitant diseases (diabetes, hypertension, dyslipidemia, gastric disorder, constipation, pain)	1.47	0.14	10.17	<0.001	4.36 (3.28–5.79)
Concomitant drug (acetaminophen, amlodipine, lansoprazole, magnesium oxide, metoclopramide, oxycodone, pantoprazole)	1.44	0.22	6.66	<0.001	4.24 (2.77–6.48)

**TABLE 4:** Multifactorial logistic analysis of the risk of induced ILD.

Variable	Estimate	Std. Error	z value	*P*	OR (95% CI)
Age	0.02	0.01	3.31	<0.001	1.02 (1.01–1.03)
Weight	−0.03	0.01	−6.58	<0.001	0.97 (0.96–0.98)
Sex Female	0.06	1.04	0.06	0.953	1.06 (0.14–8.2)
Male	0.88	1.04	0.84	0.402	2.4 (0.31–18.56)
Diabetes	−0.2	0.76	−0.27	0.787	0.82 (0.19–3.58)
Hypertension	0.64	0.39	1.62	0.105	1.89 (0.88–4.07)
Dyslipidemia	1.98	0.83	2.38	0.017	7.23 (1.42–36.83)
Gastric	1.06	1.28	0.83	0.408	2.88 (0.24–35.26)
Disorder	0.76	0.64	1.19	0.233	2.13 (0.61–7.42)
Constipation	0.56	0.68	0.82	0.412	1.75 (0.46–6.64)
Pain	0.55	0.39	1.4	0.161	1.73 (0.8–3.75)
Acetaminophen	0.2	1.05	0.19	0.851	1.22 (0.16–9.57)
Amlodipine	0.98	0.66	1.48	0.139	2.65 (0.73–9.64)
Lansoprazole	2.17	0.83	2.61	0.009	8.75 (1.72–44.52)
Magnesium oxide	0.43	0.83	0.52	0.602	1.54 (0.3–7.88)
Metoclopramide	1.07	1.13	0.95	0.342	2.93 (0.32–26.8)
Oxycodone	−11.92	288.52	−0.04	0.967	0 (0–0)
Pantoprazole	1.59	1.11	1.43	0.152	4.9 (0.56–42.91)

## 4 Discussion

ILD, characterized by alveolitis, interstitial inflammation, and pulmonary fibrosis, is an umbrella term for a range of diseases with similar symptoms (Wijsenbeek et al., 2022). Drug-induced ILD accounts for approximately 5% of all ILD cases, while ILD induced by targeted drug therapy is rarer and may be life-threatening when it occurs (Skeoch et al., 2018). In recent years, studies have reported cases of ILD associated with EGFR-TKIs (Huang et al., 2020; Shi et al., 2025; Oshima et al., 2018); however, research on EGFR-TKIs for NSCLC-induced ILD remains limited. Therefore, it is important to explore the characteristics and risks of NSCLC-associated ILD with EGFR-TKI therapy using large-scale real-world data.

EGFR-TKI has become the standard treatment for NSCLC. Our study found that most patients who developed ILD were in the elderly population, specifically those over 65 years of age. This is consistent with previous findings (Shi et al., 2025) that elderly patients suffer from comorbidities and are on polypharmacy, which increases EGFR-TKI-mediated pulmonary toxicity during long-term treatment (Decoster and Schallier, 2019). Notably, the results of the multivariate regression analysis suggest that weight gain may reduce the risk of developing ILD. However, this finding contradicts another study, which demonstrated that being overweight and obesity are associated with poorer pulmonary function (Schaeffer et al., 2022). It is important to note that our study was limited by a high rate of missing data, ranging from 22.7% to 55.9%, which to some extent compromises the reliability of the conclusion. Therefore, further research is warranted to validate the association between weight gain and the risk of ILD.

In this study, the association between EGFR-TKIs and ILD, along with its subtypes, was systematically characterized through real-world pharmacovigilance data analysis. Notably, all EGFR-TKIs showed a significant ILD signal (ROR = 24.61), a result that differs from the 0.6%–2.2% incidence reported in previous clinical trials (Takeda et al., 2015), suggesting that the occurrence of ILD may be grossly underestimated in real-world settings. In further investigation into the factors affecting EGFR-TKI-associated ILD revealed a significant correlation between ILD occurrence and concomitant dyslipidemia in patients treated with EGFR-TKI. This phenomenon may be due to the activation of lung macrophages by free fatty acids via the TLR4/NF-κB pathway, which in turn promotes their polarization towards the M1 phenotype (Suganami et al., 2007).

Furthermore, existing evidence indicates that patients receiving lansoprazole therapy have an increased risk of developing ILD (Wang et al., 2025). This trend has been corroborated by a study from Kambara et al., whose results demonstrated that patients treated with proton pump inhibitors, including lansoprazole, exhibited significantly higher rates and risks of acute exacerbation of ILD (Sato et al., 2022). Our study further reveals that the concomitant use of an EGFR-TKI with lansoprazole further elevates the risk of ILD. This phenomenon may be attributed to a drug-drug interaction involving metabolic pathways. Both EGFR-TKIs and lansoprazole are metabolized by the CYP3A4 enzyme. However, EGFR-TKIs can inhibit the activity of CYP3A4, which in turn potentiates the acid-suppressive effect of lansoprazole, thereby potentially increasing the risk of ILD (Wang et al., 2025). These findings underscore the critical need for clinicians to focus on these interactions in clinical practice, offering a valuable reference for optimizing therapeutic strategies and mitigating the risk of ILD.

Dacomitinib (ROR = 21.39) warrants attention for its high-risk profile, the extremely small sample size (*n =* 5) precludes definitive conclusions. Further large-scale studies are needed to validate this observation. In addition, our study revealed that osimertinib was associated with the highest number of reported ILD cases. Notably, 45.5% of these patients were aged 65 or older, suggesting that the elderly population may be at an increased risk of ILD due to the widespread use of this drug. This finding is consistent with the results of a previous study (Ishibashi et al., 2023). This indicates that when treating NSCLC with EGFR-TKIs, particular attention should be paid to the occurrence of this adverse effect, especially in elderly patients.

All five EGFR-TKIs induced ILD; however, side effect profiles differed between EGFR-TKIs. Subgroup analysis revealed a significant, drug-specific toxicity profile. The association of osimertinib with rare ILD subtypes, such as sarcoidosis, fine bronchiolitis, alveolar lung disease, and diffuse alveolar hemorrhage, may be related to its enhanced lung tissue penetration (Sato et al., 2022; Forte and Sangani, 2019). Similarly, gefitinib-specific signals of radiological lung injury suggest that the choice of treatment regimen should be made with extra caution in patients with a history of radiotherapy. Of note is the strong association between dacomitinib and restrictive lung disease. Some studies have shown a poor safety profile for dacomitinib (Li et al., 2024), but there is no evidence of a significant difference between dacomitinib and other EGFR-TKIs in terms of restrictive lung disease. Therefore, enhanced dynamic monitoring of lung function during treatment should still be emphasized.

The median time to onset of ILD varies among EGFR-TKIs. Erlotinib has a median latency of up to 36.5 days, which may be related to its lower lipid solubility. This results in slower deposition of the drug in the alveolar epithelium, prolonging the time it takes for the drug to reach the threshold of toxicity in lung tissue. In contrast, the early onset of toxicity with gefitinib is associated with its unique anilino quinazoline structure, which enhances the binding affinity to the EGFR receptor in alveolar type II epithelial cells, thereby accelerating the burst release of pro-inflammatory factors IL-6 and TNF-α (Scheffler et al., 2011; Ishigu et al., 2013). Notably, despite being a third-generation EGFR-TKI, the median duration of ILD episodes with osimertinib was significantly shorter than that of erlotinib. This may be related to persistent ERK signaling inhibition triggered by its irreversible binding properties (He et al., 2021).

Beyond EGFR-TKIs, ILD is a recognized complication of immune checkpoint inhibitors (ICIs), albeit with distinct mechanisms and clinical features. While EGFR-TKI-associated ILD primarily arises from direct epithelial injury and pro-fibrotic signaling in genetically susceptible hosts (Scheffler et al., 2011; Ishigu et al., 2013), ICI-related pneumonitis stems from T-cell-mediated inflammation and dysregulated immune responses (Spagnolo et al., 2022). Clinically, ICI-pneumonitis often presents earlier (median onset: 2.1–4.6 months) than EGFR-TKI-ILD and exhibits radiographic patterns such as cryptogenic organizing pneumonia more frequently (Spagnolo et al., 2022). Management also diverges, High-dose glucocorticoids are the first-line treatment for ICI-related pneumonia, while EGFR-TKI-related ILD requires immediate discontinuation of the drug, with glucocorticoids used in combination if necessary (Ohmori et al., 2021; Skeoch et al., 2018). These distinctions underscore the importance of drug-class-specific vigilance in monitoring and intervention.

This study also has several inherent limitations. First, it was not possible to assess the true incidence of ILDs associated with drug use because FAERS safety reports do not include a denominator for drug users. Second, reports in the FAERS database are submitted voluntarily, meaning that not all adverse drug events are reported, which results in missing reports, especially for rarer or milder adverse events. Finally, the contribution of EGFR-TKIs to ILD-related deaths cannot be determined with certainty, but it warrants confirmation in a large prospective study.

## 5 Conclusion

The risk of ILD is notably higher in EGFR-TKI-treated NSCLC patients and is strongly associated with patient age, comorbidities, and the concomitant use of lansoprazole. Clinicians should carefully consider these factors when selecting a treatment regimen to minimize the incidence of ILD. Follow-up studies are necessary to further investigate effective strategies for preventing and managing EGFR-TKI-associated ILD.

## Data Availability

The original contributions presented in the study are included in the article/Supplementary Material, further inquiries can be directed to the corresponding author.
