# Supplementary material for: Risk of interstitial lung disease in non-small cell lung cancer treated with EGFR-TKI: a real-world pharmacovigilance study
Source: Front Pharmacol. 2025 Aug 29;16:1652750. doi: 10.3389/fphar.2025.1652750 (PMC12426085; doi:10.3389/fphar.2025.1652750)
Supplement: Supplementary file 1 [file Table1.docx]

**Supplementary Table 1** Summary of approved EGFR-TKIs by FDA.

| **Generic name** | **Brand name** | **Drug code** |
| --- | --- | --- |
| Gefitinib | Iressa | ZD1839 |
| Erlotinib | Tarceva | OSI-774 |
| Afatinib | Gilotrif, Afatinib Maleate | BIBW-2992 |
| Dacomitinib | Vizimpro | PF-00299804 |
| Osimertinib | Mereletinib, Tagrisso, Osimertinib mesylate, Mereletinib mesilate | AZD-9291 |

EGFR-TKI, Epidermal growth factor receptor tyrosine kinase inhibitors; FDA, Food and Drug Administration.
