# Supplementary material for: Risk of interstitial lung disease in non-small cell lung cancer treated with EGFR-TKI: a real-world pharmacovigilance study
Source: Front Pharmacol. 2025 Aug 29;16:1652750. doi: 10.3389/fphar.2025.1652750 (PMC12426085; doi:10.3389/fphar.2025.1652750)
Supplement: Supplementary file 2 [file Table2.docx]

**Supplementary Table 2** Full list of preferred terms of ILD according to standardized MedDRA Queries

| Acute interstitial pneumonitis |
| --- |
| Alveolar lung disease |
| Alveolar proteinosis |
| Alveolitis |
| Alveolitis necrotising |
| Autoimmune lung disease |
| Bronchiolitis |
| Bronchiolitis obliterans syndrome |
| Chronic graft versus host disease in lung |
| Combined pulmonary fibrosis and emphysema |
| Confirmed e-cigarette or vaping product use associated lung injury |
| Diffuse alveolar damage |
| Eosinophilia myalgia syndrome |
| Eosinophilic granulomatosis with polyangiitis |
| Eosinophilic pneumonia |
| Eosinophilic pneumonia acute |
| Eosinophilic pneumonia chronic |
| Hypersensitivity pneumonitis |
| Idiopathic interstitial pneumonia |
| Idiopathic pneumonia syndrome |
| Idiopathic pulmonary fibrosis |
| Immune-mediated lung disease |
| Interstitial lung abnormality |
| Interstitial lung disease |
| Low lung compliance |
| Lung infiltration |
| Lung opacity |
| Necrotising bronchiolitis |
| Obliterative bronchiolitis |
| Pleuroparenchymal fibroelastosis |
| Pneumonitis |
| Probable e-cigarette or vaping product use associated lung injury |
| Progressive massive fibrosis |
| Pulmonary fibrosis |
| Pulmonary necrosis |
| Pulmonary radiation injury |
| Pulmonary toxicity |
| Pulmonary vasculitis |
| Radiation alveolitis |
| Radiation bronchitis |
| Radiation fibrosis - lung |
| Radiation pneumonitis |
| Respiratory syncytial virus bronchiolitis |
| Rheumatoid arthritis-associated interstitial lung disease |
| Small airways disease |
| Transfusion-related acute lung injury |
| Acute lung injury |
| Acute respiratory distress syndrome |
| Airway remodelling |
| Allergic eosinophilia |
| Antisynthetase syndrome |
| Biopsy lung abnormal |
| Complications of transplanted lung |
| Cystic lung disease |
| Goodpasture's syndrome |
| Granulomatosis with polyangiitis |
| Granulomatous pneumonitis |
| Langerhans' cell histiocytosis |
| Loefgren syndrome |
| Lung induration |
| Lung transplant rejection |
| Lupus pneumonitis |
| Lymphangioleiomyomatosis |
| Organising pneumonia |
| Pneumonitis chemical |
| Polyarteritis nodosa |
| Pulmonary alveolar haemorrhage |
| Pulmonary bullae rupture |
| Pulmonary contusion |
| Pulmonary eosinophilia |
| Pulmonary granuloma |
| Pulmonary haemosiderosis |
| Pulmonary renal syndrome |
| Pulmonary sarcoidosis |
| Pulmonary septal thickening |
| Restrictive pulmonary disease |
| Rheumatoid lung |
| Sarcoidosis |
| Systemic sclerosis pulmonary |
| Toxic oil syndrome |
