# Supplementary material for: Risk of interstitial lung disease in non-small cell lung cancer treated with EGFR-TKI: a real-world pharmacovigilance study
Source: Front Pharmacol. 2025 Aug 29;16:1652750. doi: 10.3389/fphar.2025.1652750 (PMC12426085; doi:10.3389/fphar.2025.1652750)
Supplement: Supplementary file 3 [file Table3.docx]

**Supplementary Table 3** A two-by-two contingency table and detailed formulas for disproportionality analysis.

|  | **Target adverse drug event** | **Other adverse drug events** | **Sums** |
| --- | --- | --- | --- |
| Target drug | a | b | a+b |
| Other drugs | c | d | c+d |
| Sums | a+c | b+d | N=a+b+c+d |

| **Algorithms** | **Equation** | **Criteria** |
| --- | --- | --- |
| ROR | ROR = ad/bc | Lower limit of 95% CI > 1, N ≥ 3 |
|  | 95%CI = e^ln(ROR)±1.96(1/a+1/b+1/c+1/d)^0.5^ |  |
| PRR | PRR = [a(c+d)]/[c(a+b)] | PRR ≥ 2, χ^2^≥ 4, N ≥ 3 |
|  | χ^2^=[(ad-bc)^2](a+b+c+d)/[(a+b)(c+d)(a+c)(b+d)] |  |
| BCPNN | IC = log_2_a(a+b+c+d)/[(a+c)(a+b)] | IC025 > 0 |
|  | 95%CI = E(IC) ± 2[V(IC)]^0.5 |  |

The formulas to calculate the signal strength are as follows: a number of reports containing both the target drug and the target adverse drug event; b, number of reports containing other adverse drug events of the target drug; c, number of reports containing the target adverse drug event of other drugs; d, number of reports containing other drugs and other adverse drug events. Abbreviations: 95% CI, 95% confidence interval; N, the number of reports; χ2, chi-squared; ROR, reporting odds ratio; PRR, proportional reporting ratio; BCPNN, Bayesian confidence propagation neutral network; IC, information component; IC025, the lower limit of the 95% CI of the IC; E (IC), the IC expectations; V (IC), the variance of IC.
